# Athlete experiences of communication strategies in applied sports nutrition and future considerations for mobile app supportive solutions

**DOI:** 10.3389/fspor.2022.911412

**Published:** 2022-09-12

**Authors:** David Mark Dunne, Carmen Lefevre-Lewis, Brian Cunniffe, Samuel George Impey, David Tod, Graeme Leonard Close, James P. Morton, Rebecca Murphy

**Affiliations:** ^1^Research Institute for Sport and Exercise Sciences, Liverpool John Moores University, Liverpool, United Kingdom; ^2^Centre for Behaviour Change, University College London, London, United Kingdom; ^3^Department of Surgery, University College London, London, United Kingdom; ^4^English Institute of Sport, Manchester, United Kingdom; ^5^Centre for Exercise and Sports Science Research, School of Medical and Health Sciences, Edith Cowan University, Joondalup, WA, Australia

**Keywords:** nutrition, behavior, mobile, technology, performance

## Abstract

**Aim:**

This study aimed to explore athletes' experiences and opinions of communication strategies in applied sports nutrition, as well as capture suggestions for future mobile app supportive solutions.

**Methods:**

A qualitative approach was used for this research. Data was generated from semi-structured focus groups (*n* = 9) with a purposive sample of 41 (male = 24, female = 17) full time professional athletes (mean age 24 ± 4.59) from five sports (football, rugby union, athletics, cycling, and boxing). Data was analyzed using reflexive thematic analysis.

**Results:**

The analysis identified four higher order themes and five sub themes. Athletes appear dissatisfied with the levels of personalization in the nutrition support they receive. Limited practitioner contact time was suggested as a contributing factor to this problem. Athletes acknowledged the usefulness of online remote nutrition support and reported a desire for more personalized technology that can tailor support to their individual needs.

**Conclusion:**

Athletes experienced a hybrid human-computer approach that combines in-person and remote digital methods to communicate with and receive information from practitioners. Mobile technology may now afford sports nutritionists with new opportunities to develop scalable solutions to support practice.

## Introduction

The daily nutritional practices of an athlete can influence not only how their body adapts to a training stimulus and performs in competition, but also how their body maintains immune function and supports general health (Close et al., 2016; Impey et al., 2018; Walsh, 2019). Dietary strategies have been developed during the last 50 years to optimize the type, timing and total amounts of foods, fluids and ergogenic aids that an athlete may consume (Thomas et al., 2016). More recently, between 2012 and 2018, sports nutrition has experienced a 4-fold increase in the number of research papers published making it one of the fastest growing and evolving disciplines in sports and exercise science (Close et al., 2019). This rapid rise in research is reflected in the applied setting where it is now common practice for sports teams, organizations and institutes to employ sports nutritionists on a part-time, full-time or consultancy basis.

The growing popularity of applied sports nutrition has also coincided with the emergence and uptake of Web 2.0's novel digital technologies (McGee and Begg, 2008). On a global scale, these social platforms, such as Facebook, WhatsApp, Instagram and Twitter, have changed how we communicate, as well as how we generate, access and consume content (Gagnon and Sabus, 2014). Practitioners have been encouraged to embrace these tools and consider their use for intervention delivery and service provision in applied settings (Ahmed et al., 2015). Applied sports nutrition has demonstrated an uptake of these digital communication tools in practice where their implementation has been deemed beneficial by applied practitioners (Dunne et al., 2019).

Despite the rapid uptake of novel digital technologies by practitioners, and increased publication of sports nutrition research over the past decade, there remains a distinct absence of implementation science research exploring the application of such tools in the sports nutrition literature. Instead, position stands and practical recommendations remain focused on increasing our understanding of biochemistry, physiology and physical performance, resulting in improvements in knowledge (Close et al., 2016; Thomas et al., 2016; Stellingwerff et al., 2019a; Collins et al., 2020). This lack of implementation research in the sports nutrition field may now be impeding the application of the progress made in the laboratory (Eccles and Mittman, 2006; Bentley et al., 2020). Instead, applied intervention studies remain focused on education despite an awareness that the translation of knowledge into nutrition behaviors in athletes remains imperfect (Heaney et al., 2011; Bentley et al., 2021; Foo et al., 2021; Tam et al., 2021).

As sports nutrition research continues to develop and the use of technology continues to permeate practice, service provision may now benefit from increasing its understanding of how athletes experience the communication strategies employed by a practitioner in applied sports nutrition. These reported experiences may help practitioners identify areas for improvement in practice; determine any current or potential future problems; enable practitioners to better target the use of their time when providing support to athletes, organizations and institutions; and may support the development of innovative ideas for delivery (Crawford, 2002).

Using qualitative methods, this study aimed to explore athletes' experiences and opinions of communication strategies in applied sports nutrition, as well as their suggestions for future mobile app supportive solutions. This acquired understanding of how athletes experience and think about the support they receive, as well as their suggestions for technology, will contribute to development of new and improved applied service provision strategies.

## Materials and methods

### Overall study design

This study is part of a program of research exploring the use of, and opportunities for, technology delivered digital interventions to improve athletes' dietary behaviors and nutritional adherence. Phase 1 focused on practitioners and involved exploratory online surveys and interviews to investigate sports nutritionists (*n* = 44) current use of digital platforms as part of nutrition service provision, as well as to identify the barriers and enablers to using such technologies from a practitioner's perspective (Dunne et al., 2019). Gathering this information from a practitioner's point of view helped identify and frame questions for the following phase. Phase 2 presented in this current study, focuses on athletes, and qualitatively captures their experiences and opinions of present-day communication strategies in applied sports nutrition, as well as their suggestions for future mobile app supportive solutions. Qualitative data was generated from semi-structured focus groups with open ended questions, similar to Bentley et al. (2021). Additional following phases of this program of research are to be determined following the combined analysis of the outcomes of phases 1 and 2.

### Participants

A purposive sampling approach was used to identify athletes from high performance sport that met the following inclusion criteria: (a) >16 years of age, and (b) were classified as tier 3 or above according to the 6-tiered Participant Classification Framework (McKay et al., 2022). The Participation Classification Framework uses training volume and performance metrics to classify participants to one of the following: Tier 0: Sedentary; Tier 1: Recreationally Active; Tier 2: Trained/Developmental; Tier 3: Highly Trained/National Level; Tier 4: Elite/International Level; or Tier 5: World Class. An initial e-mail describing the study was distributed to a variety of sport science and medicine practitioners working in UK high performance sport. Practitioners volunteered as gatekeepers at their sporting organizations, inviting athletes to participate and helping arrange focus group dates and times for the interested parties. Nine groups of athletes (*n* = 41; male = 24, female = 17; mean = 6; range = 3 to 8) from five sports (football *n* = 21, rugby union *n* = 8, athletics *n* = 3, cycling *n* = 4, and boxing *n* = 5) were recruited to participate in this qualitative study. Of the intermittent field sports (football and rugby union), 52% of athletes were classified as tier 3, 27% were classified as tier 4, and the remaining 21% of athletes were classified as tier 5. In the remaining sports (athletics, cycling and boxing), 83% of athletes were classified as tier 4, with the remaining 17% of athletes classified as tier 5. All participants were full time professional athletes with an average age of 24 years (SD = 4.59). The current level of nutrition service provision received by the participants varied from 2 days per month consultancy up to full time support. All participants reported receiving a minimum of 2 years and a maximum of 15 years of nutrition service provision as part of their sporting careers to date (average 6.6 years, SD = 4.11).

### Procedures

This qualitative study used focus groups, designed and reported in line with the Consolidated Criteria for Reporting Qualitative Research (COREQ) (Tong et al., 2007). Focus group interview guides were developed by DD and RM to explore participants experiences and opinions of communication strategies in applied sports nutrition, as well as their suggestions for future digital technology supportive solutions. The questions devised were open ended and supported by a range of additional prompts to probe for further explanation (see Appendix 1). Semi-structured interviewing techniques allowed for in-depth exploration of the topics in a flexible but consistent manner (Sparkes and Smith, 2014). This approach ensured participants had the opportunity to share their own thoughts and feelings toward to the topics. The interview questions were piloted with a small sample (*n* = 4) of tier 3 and tier 4 athletes prior to data collection. One question was removed following this pilot and athlete feedback to avoid repetition. No data generated from the pilot was included in the final analysis.

All focus groups were facilitated by one moderator (DD), who was trained in qualitative research methods. The composition of these focus groups was largely dictated to by athlete availability around their training and competition schedules. However, efforts were made with the gatekeeper to select participants of different ages to encourage different points of view, as well participants with different degrees of experience with nutrition support to avoid groupthink. To generate rich interactions DD played an active role in facilitating the group discussions and efforts to establish good rapport were made with the participants throughout (Tausch and Menold, 2016). Each focus group was individually adapted to the flow of discussion taking place. Planned as well as naturally occurring “probing” questions were used to add further depth, context and insight to the responses from participants (Gratton and Jones, 2004; Turner, 2010; Sparkes and Smith, 2014). To operationalize, DD directed follow up questions and probes in response to other participants initial answers to specific individuals at various timepoints to ensure there was a variety of participants experiences and opinions captured during this process. Focus groups were deemed suitable for this exploratory research due to the spontaneous, expressive and emotional interaction they can generate as participants are able to respond to and build upon one another's comments, stimulating a breadth of discussion (Wong, 2008; Sparkes and Smith, 2014). Additionally, focus groups can challenge and develop an individual's viewpoint and provides the opportunity for norms and assumptions to be revealed (Kitzinger, 1995). Focus groups were carried out face to face to promote participation and took place across a range of UK based training centers (Tausch and Menold, 2016). Written consent was obtained from participants prior to each focus group commencing. Focus groups lasted an average of 27 min (SD = 7) and were recorded using a handheld audio recording device (Tascam DR-05X). Field notes and a research journal were kept during data collection.

### Data analysis

Given that the researcher's role is vital in knowledge production, a reflexive thematic analysis (TA) approach was implemented (Braun and Clarke, 2019). Reflexive TA facilitated a richer and more nuanced reading of the data as it required the researchers to continually question and query any assumptions made during the interpretation and coding of the data (Braun and Clarke, 2019). To identify and construct patterns of meaning from the data, the analysis followed a six-stage approach (Braun and Clarke, 2006). Initially the audio recordings were listened to multiple times before being transcribed verbatim by DD. Familiarization with the data (Stage 1) continued as transcripts were repeated read and initial notes for coding were made. Following familiarization, initial codes were generated inductively (Stage 2) using NVivo 11 software. Stage 3 involved organizing codes into the following four semantic themes: (1) communication strategies and information delivery, (2) acceptance and adoption of the online practitioner, (3) a personalization problem and (4) preferred mobile app features. Themes were then reviewed by authors (Stage 4) to check and challenge any assumptions made by DD during the interpretation of the data. A thematic map was used to reflect the meanings evident in the data set as a whole. Themes were then defined and refined (Stage 5) to ensure they individually captured the essence of what each theme was about. As a result, theme 4 was renamed “tailoring technology” and new sub themes developed: (4a) periodized and personalized nutrition planning, (4b) feedback loops, (4c) nudges, and (4d) performance focused content. For the final stage (Stage 6), an analytic narrative was produced and is presented in this manuscript. Throughout, pseudonyms were assigned to participants to protect their identity. Member reflections were carried out by DD with a selection (*n* = 9) of participants (selected based upon availability) to generate additional data and insights, as well as to explore any gaps in the results and concerns the participants had over the interpretations of the findings (Braun and Clarke, 2013; Smith and McGannon, 2017).

## Results

The purpose of this study was to explore athletes' experiences and opinions of present-day communication strategies in elite sports nutrition, as well as their suggestions for future mobile app supportive solutions. The focus group analysis identified a total of four higher order themes and five sub themes. A detailed description of each theme and associated sub theme is outlined in this section with evidence in the form of indicative verbatim quotations to highlight the participants narrative. A summary of these findings is presented in Table 1. Athletes experienced a hybrid human-computer approach to sports nutrition support, whereby practitioners employed a range of in-person and remote digital methods, to communicate with and deliver information to athletes. Athletes appeared unsatisfied with the current nutrition support they received. Lack of personalization and limited contact time with practitioners were highlighted as contributing to this feeling of discontentment. Despite perceptions of limited contact time, athletes acknowledged the usefulness of receiving remote nutrition support, and reported a general acceptance and adoption of this online service. Regarding mobile app supportive solutions, athletes identified an opportunity for the introduction of tailoring technology to help them periodize and plan their nutrition in line with the demands of their activity. Supportive features to help drive engagement and self-monitoring were also suggested by athletes as being useful.

**Table 1:** A summary table of identified themes.

**Raw data**	**Sub theme**	**Higher order theme**
“*The nutritionist is here most of the time, but I probably only really speak to them seriously about nutrition maybe once every 3 weeks. That would be about right. I think it's got better this year. I think last year you could not have really had a proper conversation for a couple of months at a time”* (Athlete 1, Focus Group 2)		Communication strategies and information delivery *(n = 33)*
“*having WhatsApp conversations are handy because you can literally send a picture or have a chat about a recipe or something quite quickly”* (Athlete 1, Focus Group 8)		Acceptance and adoption of the online practitioner *(n = 26)*
“*I think what the nutritionist does is pretty much pointless I'd say. It should be related to exactly what your training is, and it should be completely personal. Unless it's every day with your training and then related to that it's pointless* (Athlete 2, Focus Group 5)	*Lack of personalization (n = 32)*	A personalization problem *(n = 47)*
“*I think just because of the limited time the nutrition support is quite generic... it is better to have a bit more input on an individual basis”* (Athlete 2, Focus Group 1)	*Limited contact time (n = 17)*	
“It would be great if you had an app where you could write ‘right, this is what I'm doing this week, we are on our training programs' and then if they said ‘right, this is how many macros you need' or whatever, for that workout for that day and week and if you're not doing that much, ‘this is how much, how many calories you need and have it all been broken down'. So, flipping it on its head with inputting training and then knowing what to eat” *(Athlete 5, Focus Group 1)*	*Periodized and personalized nutrition plans (n =36)*	Tailoring technology *(n = 54)*
“*Even in an app, inputting a bit of personal information would be useful, so you can actually track your weight and record things so you can see if it is actually making a difference...So say if you are 70 kg on this date and you use this and you can actually see a difference, ‘oh I'm actually 68.4 now' and you can see that”* (Athlete 2, Focus Group 6)	*Goal setting, monitoring and feedback (n = 14)*	
“*alerts and stuff like that would be helpful, different things to keep you engaged with the app. Following a path, you know, would be good.”* (Athlete 4, Focus Group 6)	*Notifications and reminders (n = 7)*	
“I think that an app should be in detail and might have at the start be quite simple so that everyone understands and then maybe underneath you might have the more complicated details of it because if you really know what you want to do or what you're eating things for then that would be the reason why.” *(Athlete 2, Focus Group 7)*	*Performance focused content (n = 8)*	

### Theme 1: Communication strategies and information delivery

Athletes discussed a range of communication strategies employed by sports nutritionists to deliver information. The strategies described included a range of traditional nutrition education methods such as group presentations. To illustrate, Jill shared how “*the nutritionist gives us as Powerpoint on the basics we need to know, like carbs, the intakes and stuff”*. The delivery of these presentations appeared to be more front loaded in the athletes sporting calendar year as exemplified by Richard who highlighted how they “*had some nutrition at the start of the year on training camp, we did some tests and went over a few things there in a bit of a presentation and a similar thing in February, and it's just mainly going through what you'd do on different days in terms of how much training you're doing.”* However, athletes did convey frustration toward the content delivered during their discussions, for instance Pete acknowledged that what they were receiving was “*the same Powerpoints that we've been seeing for quite a few years”* whereas Ben described the content as “*just very basic.”*

The focus groups also generated patterns of talk around the athlete's individual experiences of one-to-one nutrition consultations. Monica described how “*they (the nutritionist) were thorough so that's why the contact time wasn't regular, because you did take away quite a lot of information from one, but it was sometimes a bit overwhelming”*. Some athletes highlighted the triggers that lead to a consultation, for instance Mike said “*if you ask to see the nutritionist, it's whenever you're injured. They'll say, “we'll have a little meeting” but then they'll give you stuff and I think everyone had a meeting in pre-season to go over everything.”* However, the frequency of the consultations appeared to vary between groups, as well as within groups year to year. Josh shared how “*the nutritionist is here most of the time, but I probably only really speak to them seriously about nutrition maybe once every 3 weeks”* before elaborating how this was an improvement from the previous year when “*last year you could have not really had a proper conversation for a couple of months at a time.”* Similar to Josh's current experience, Julie also shared how “*the nutritionist is not in all the time but when they are in, it's like once a week...I say, we have a meeting once a month.”* Emily described some potential barriers that may be limiting athletes one-to-one consultations opportunities:

“*the nutritionist is available to talk to but obviously it's limited contact time. They give us talks when we're in (international) camp... they're not always at every camp. And obviously if they're there and everyone's trying to get some input from them, you can't sit down for an hour and discuss things.“*

Notably, some athletes experience support *via* unstructured and informal conversations (commonly known in the domain as “corridor conversations”). This approach was a particularly valuable communication strategy that enabled the athlete to share and receive information quickly. To illustrate, Frank shared:

“*I think by having a nutritionist here all the time is easier because then you can just grab them in passing and be like ‘this is what I've eaten. Quite often when I weigh in, in the morning we'll chat about what I've eaten over the last day and why I'm heavy or why I've lost weight and where I can look at targeting to help put that on. I think that's been really good.”*

In addition to face-to-face methods of communication, several digital strategies were also described by the athletes, of which the use of WhatsApp was the most discussed. For example, Monica shared how “*having WhatsApp conversations are handy because you can literally send a picture or have a chat about a recipe or something quite quickly.”* This ability to get feedback quickly was also highlighted by Jack who shared “*if we've got a question the nutritionist will reply within an hour or something.”* In addition, Roy described the level of support being provided by sports nutritionists over this particular digital platform “*WhatsApping, there's loads and loads of nutrition support.”* Mike further elaborated on this to illustrate how practitioners were using this communication channel in their applied practice:

“*There's a WhatsApp group for nutrition…the nutritionist will put loads of stuff in, like some days they'll say there's going to be an update on what's going to be in the canteen that day, on what type of thing you can eat and what type of day you've had and if there's a game they'll say what you should be eating.”*

However, WhatsApp was not the only digital platform described as being used by sports nutritionists to deliver information to athletes, for example Josh tell us how they “*spoke to the nutritionist on Instagram and got a few things which I felt like I was lacking”* before further detailing how their increased likelihood to engage with this content in comparison with email “*I think there will be more chance of them picking it up than an e-mail.”*

Non-social media digital communication strategies were also described by athletes as a means to receive feedback from the nutritionist. The use of a range of purpose-built nutrition apps and how the athletes engage with them was described by Rachel:

“*We used an app (Meal Logger) which I took pictures of my food and we'd send it to the nutritionist every day. The nutritionist was just seeing how I was eating, what I was eating, when I was eating and then I'd use another app (MyFitnessPal) which scanned bar codes of whatever you were eating and whatever you were making and you'd put it in and it'd count your calories.”*

These experiences and insights from athletes illustrate the variety of methods employed by the modern sports nutritionist in an attempt to communicate with, and deliver information to, the athletes they may be working to support.

### Theme 2: Acceptance and adoption of the online practitioner

Some athletes described how the nutrition support they now receive from a practitioner had moved to more of an online format. For instance, Susan said “*it (the nutrition support) was basically working so that I could get in touch with the nutritionist over WhatsApp if I had any concerns.”* This remote online approach was deemed useful by athletes, as illustrated by Elizabeth, “*the nutritionist is always at the end of a phone or a WhatsApp, which is really handy.”* Athletes highlighted the increase in accessibility as a potential driver of this uptake, as discussed by Richard saying, “*I think using apps are ideal really because everyone's on their phone aren't they.”* Some athletes also suggested this remote service can solve logistical issues athletes face and be complementary to on-site support. Typifying this is Josh:

“*I think, in passing it's easy at the club but having an app is much easier. You can just be like, ‘boom' rather than be like ‘come and I'll see you at this time' and you're ‘well actually I can't see you at that time' or you've got to change everything around it. It's hard enough anyway when you're trying to book in to see a coach or something”*

The types of interactions athletes had with practitioners and the resources they received *via* digital platforms varied amongst the athlete groups. Some athletes detailed more of a check in support service, such as Monica who said, “*we've had WhatsApp conversations when I've been in America, just to check, on a few of the things that we've agreed to do.”* Others highlighted how the online environment has become more of a document sharing platform, as discussed by Emma, “*We've had stuff sent on WhatsApp which helps…PDF documents and nutrition plans.”*

However, despite the widespread acceptance and adoption across the majority of the athlete groups, access to online support was not uniform across all sports. Despite an appetite for the online service, some focus group discussions identified its absence. To illustrate, Erica said:

“*I think that online support would be a game changer… players probably do want to ask questions and if you do ask a sports scientist sometimes, they don't actually have the nutritionist answer”*.

These athlete insights illustrate the general acceptance and adoption of an online practitioner service to providing sports nutrition support. However, the delivery of service to athletes currently appears to vary greatly.

### Theme 3: A personalization problem

Athletes described a lack of personalization in the nutrition support they received both in person and digitally. For example, Rachel described how “*it (the nutrition support) was only in terms of ideas really, but it's not really player specific stuff.”* This experience was consistent across the groups and is further illustrated by Jill who described how “*there's a basic structure there but there's nothing, I wouldn't say, in-depth or anything.”* Some athletes described this absence of personalization in more detail and highlighted areas that they perceived could add value. To illustrate, Charlie discussed the usefulness, and absence of, receiving individualized macronutrient requirements and targets “*I follow a macro specific diet and found that really worked but we don't get offered anything in that much detail.”* Not all athletes described a need for this level of detail but there was strong agreement that some level of tailored nutrition planning would be valuable. Exemplifying this is Ross who said, “*If you could narrow it (a nutrition plan) down to your personal needs then it would be beneficial.”*

This overall absence of personalization led to significant frustration in a number of athletes and led them to question the usefulness of this generic approach to service provision. To illustrate Jack said:

“*I think what the nutritionist does is pretty much pointless I'd say. It should be related to exactly what your training is, and it should be completely personal. Unless it's every day with your training and then related to that, it's pointless.”*

These comments demonstrated an understanding from the athletes of why the nutrition support they receive may be the way it is currently. Most notably, athletes suggested that the problems they identified may be the result of limited practitioner contact time. For instance, Emily acknowledged that “*because of the limited time, the nutrition support is quite generic”* as well as highlighting the part time nature of the sports nutrition service provision “*They give us talks when they're (the nutritionist) in camp…they're not at every camp.”* Judy revealed other reasons why contact time maybe limited, suggesting that the problem stems from having to service large squad numbers “*I think it's hard because the nutritionist has got to do the whole squad so they can't just individualize it for everyone.”* This resonated with other athletes and is described by Ben who said, “*you can't individualize for all of us.”*

The combination of limited contact time and an absence of personalization resulted in some athletes taking matters into their own hands as described by Mike, “*If I had to know something, sometimes I just Google it and get the answer quite easily.”* These experiences of the athletes illustrate not only the challenges they are facing as individuals, but also the practical issues facing practitioners, such as time and scale.

### Theme 4: Tailoring technology

Athletes described a desire for technology that could tailor their nutrition according to their training demands. For instance, this was highlighted by Charlie who said:

“*It would be great if you had an app where you could write ‘right, this is what I'm doing this week, we are on our training programs' and then if they said ‘right, this is how many macros you need' or whatever, for that workout for that day and week and if you're not doing that much, ‘this is how much, how many calories you need and have it all been broken down'. So, flipping it on its head with inputting training and then knowing what to eat”*

This resonated with Phoebe who shared “*What would be cool is if you could do something based on what training you put in and what you should be eating”* before further elaborating to say why they felt this is should be the preferred approach, “*because some days you double run and some days you do gym sessions and running and it's, the sports are different as well.”* There was strong agreement for this rationale among the athlete groups. Typifying this was Ben who highlighted that their nutrition needs “*depends on how active we are.”*

### Periodized and personalized nutrition planning

The athletes drew on their previous experiences and exposure to nutrition interventions to guide suggestions for the technology that they believed would be beneficial. The most prevalent suggestion that echoed throughout the majority of the athlete groups was the usefulness of periodized and personalized nutrition planning. To elaborate, Frank said:

“*Those carb periodization frameworks would be useful and I think with recipes that go with it. So, if you are saying something like a low carb or something like that, just be like ‘this is a great option, this is easy to do, boom, there's the recipe.”*

Notably, some athletes described how in the future this type of technology may be available to empower them, as illustrated by Barry saying:

“*Maybe someday I'll probably be using an app just to, you know, because you can see, if you've had a hard day, what you could see what sort of things you should eat. You just give it the information and it makes a decision for you.”*

Athletes also described how these periodized plans could be made more interactive to supply them with more recipe ideas, such as Emma who said, “*you can link a color coded plan to a video of a high carb meal that you could have match day*−*1 or something.”* This simplicity of delivery using color coding was highlighted as being important and is discussed by Amy, “*It seems easier if you know what color food you are.”* Athletes drew reference to other technologies they currently use to illustrate the value of a solution with a simple design, as described by Richard:

“*The thing I find quite important, and I think Training Peaks do that quite well, when you've got your week up and then you can have a look at a weekly snapshot and stuff. So yeah, I suppose the layout is quite important (for nutrition plans).”*

### Performance focused content

Athletes described a need for content that could provide a rationale for their plan. Elizabeth discusses this saying “*I would like to have a bit of a reason why you're doing the nutrition plan.”* Some athletes commented that technology that could deliver this performance focused content would help drive their engagement with nutrition. For example, Joey shared:

“*I bet there's loads of knowledge out there that certain foods help you in different situations, ‘if you're sore this food will help me for this', ‘I've got a really hard training session coming up, I need to be lighter', ‘this would be the correct food to have', do you know what I mean? If an app had that sort of knowledge, I would use it every single day, yeah, every day.”*

The focus group discussions generated patterns of talk around this need for supporting information which could help provide clarity and confidence in their proposed nutrition plan.

### Feedback loops

The ability for technology to support self-monitoring was also suggested as being useful. Athletes described how these features could provide feedback loops that would enable them to quantify their progression or regression. Charlie illustrates this point when discussing tracking weight related data:

“*Even in an app, inputting a bit of personal information would be useful, so you can actually track your weight and record things so you can see if it is actually making a difference...So say if you are 70 kg on this date and you use this and you can actually see a difference, ‘oh I'm actually 68.4 now' and you can see that.”*

Again, athletes drew on their experiences with previous technology to describe how these features may be presented visually. Jack shared how, similar to Training Peaks, “*you can have graphs and see how're you're doing.”* A range of feedback loops were identified in discussions with athletes and included monitoring adherence to a nutrition plan, sometimes by tracking macronutrient intake, as well as tracking progress against a goal or a challenge which may or may not be weight related.

### Nudges

Athletes discussed how technology features such as notifications or nudges could help to support their engagement with technology. Exemplifying this is Chandler who said “*alerts and stuff like that would be helpful, different things to keep you engaged with the app. Following a path, you know, would be good.”* Similarly, Josh shared how notifications may help to prompt behaviors such as cooking:

“*I think, to be honest, maybe, for me, a reminder, you know it could notify you because, like on days off especially, I can go through and I can be hungry but not cook because I can't be bothered to get out of bed and that's genuine.”*

These athlete experiences and insights help to illustrate the desire for a nutrition tailoring technology, as well as provide insight into what sort of features a potential future solution may have.

## Discussion

This study used qualitative research methods to explore athletes' experiences and opinions of communication strategies in applied sports nutrition, as well as their suggestions for future mobile app supportive solutions. The findings revealed that athletes experience a hybrid human-computer approach to nutrition support from practitioners. Group presentations, one-to-one consultations and “corridor conversations” were the most prominent in-person communication strategies employed by sports nutritionists, although the frequency of these events and athletes' satisfaction appeared to vary. Digitally, the use of social media platforms and mobile applications was common across the majority of group as athletes accepted and adopted the online practitioner. Additionally, it was identified that athletes perceived a lack of personalization and expressed a desire for individual tailoring in the applied sports nutrition support they currently receive. Finally, a desire for tailoring technology that could provide athletes with periodized nutrition plans tailored to the demands of their training and competing, performance focused content, feedback and nudges was also reported.

This research is the first to identify that elite athletes, across a variety of sports, perceive a lack of personalization in the applied sports nutrition support they receive. Findings outline a desire among athletes for more tailored nutrition provision. This is consistent with the demands of the general population who report a need for more individualized nutrition care in clinical settings (Sladdin et al., 2017). Despite this desire, the majority of research efforts in sports nutrition to date have focused on increasing our understanding of how nutrient availability modulates metabolism and physiology (Jonvik et al., 2022). These efforts have led to the growth and evolvement of strategies such as nutritional periodization which has rapidly become a hot topic in sports nutrition literature (Jeukendrup, 2017; Burke and Hawley, 2018; Stellingwerff et al., 2019b). However, the optimal delivery of such a nuanced intervention that requires a practitioner to be adequately trained in the physiology of training has yet to be explored. As this area has yet to be investigated in sports nutrition, the possibility and potential of delivering the athletes desired level of personalisation is unknown. As a result, what athletes want and what practitioners can deliver may require further attention and more critical thought.

In contrast to sports nutrition, clinical fields of practice, such as obesity and diabetes management, have dedicated time and resource to improving the design and delivery of tailored interventions and “precision” initiatives that utilize technology to progressively move toward patient support that is more individualized, contextualized and timely (Chevance et al., 2020; Craig et al., 2020). These personalized interventions have been shown to produce significantly stronger health outcomes in both general and clinical populations across a range of variety of health behaviors including, but not limited to, diet and nutrition when compared with more static traditional approaches (Wang and Miller, 2019; Craig et al., 2020). To elaborate further, the athletes in this research describe the frequency at which they speak to a nutritionist “*seriously*” as every 3–4 weeks, yet also highlight that they communicate with their practitioners informally and/or digitally on a more frequent basis in an attempt to get feedback or request a resource. In these scenarios data is harvested by practitioners from a single timepoint (e.g., during a conversation, or *via* a series of WhatsApp messages, etc) as they make a static assessment and determine *how* or *what* is delivered. These decisions often rely on tacit knowledge which can vary from practitioner to practitioner depending on their level of applied experience, as is the case with other fields of practice (Gertler, 2003). As a result, these static approaches may be subject to high degrees of variability between practitioners. Although standardizing training may help reduce this variation it is unlikely to compensate for the multiple additional years of applied experience a senior practitioner may have over a neophyte practitioner. However, technology enabled interventions, such as adaptive and continuous tuning interventions, have shown promise to support a more dynamic approach, where data can be harvested from multiple timepoints to feed algorithms that refine the intervention content, delivery or timing to the idiosyncrasies of an individual (Almirall et al., 2014; Hardeman et al., 2019; Huckvale et al., 2019; Chevance et al., 2020). These novel and emerging methodologies may now provide sports nutrition academics and practitioners an opportunity to optimize the tailoring of communication and intervention delivery strategies and become “early adopters” of technology advancements that may accelerate the evolvement of their hybrid human-computer approach (Nahum-Shani et al., 2017; Dearing and Cox, 2018; Huckvale et al., 2019).

The findings of this research also identified that, despite practitioners now being available to communicate with athletes online as well as in person, athletes perceived that practitioner time and resources may be spread too thinly across organizations and be a contributing factor to the lack of personalization they experience. These suggestions are corroborated by Dunne et al. (2019) who found that, on average, sports nutritionists reported working across three different sports reflecting the part time and consultancy nature of the profession. An industry shift toward more full-time sports nutrition employment may support an improvement this situation, however more full-time roles alone will not completely resolve this issue as a single practitioner can still face squad sizes of up to 64 individual athletes in one organization, as demonstrated by the UK's rugby union premiership in 2019–20 (Shaw, 2019). Sports nutrition may now need to solve for scale and consider implementing solutions that have the potential to reach large numbers of people in a time and cost-effective manner to support practitioners, similar to other sectors of the healthcare industry where mobile health (mhealth) initiatives have transformed clinical practice (Steinhubl et al., 2015; Vandelanotte et al., 2016). Deloitte (2020) highlighted that digital technology solutions led to a 60% reduction in paperwork time and a 29% increase in patient face time for community nurses, as well as cost savings of 40% compared with usual care within the UK National Health Service. These trends are consistent across the modern healthcare system as it transitions to one that is more participatory and personalized (Goetz and Schork, 2018; Johnson et al., 2020). These advancements in scalable technology solutions rely on algorithms that follow a set of processes to achieve a certain result. Given this, perhaps a consideration for sports nutritionists now is to identify what of their roles may be best suited to being outsourced to technology and what remains heuristic thinking. It is worth noting, however, that as these questions are answered and the advancements in implementation science are applied, sports nutritionists' traditional roles may be modified and new opportunities for employment may arise within this space (Masys, 2002).

Although technology appears to hold multiple potential communication and intervention delivery solutions and opportunities for athletes and practitioners, proceeding with an agnostic view may be best suited to the rapidly evolving digital landscape. How, when and where an individual's physiological data can now be captured, interpreted and returned is no longer limited to lab-based settings (Plews et al., 2017; Falter et al., 2019; Miller et al., 2021). Instead, mobile phones, smart watches and biometric rings (e.g., Apple Watch, WHOOP and Oura Ring) are now demonstrating efficacy in the remote capture of continuous data and the delivery of app-based interventions that leverage principles from health behavior theories to improve health and performance behaviors such as sleep (Reeder and David, 2016; Browne et al., 2021). These experiences are currently limited by hardware, e.g., mobile phones, however the development of web 3.0, augmented reality and the metaverse may create new highly immersive environments for practitioners to create, share, educate and influence through virtualisation (Kye et al., 2021). Echoing the work of Jonvik et al. (2022), it does appear applied sports nutrition is at a critical juncture in its evolution and is primed to utilize new technologies to support athletes.

## Limitations

No practitioner data was captured during this study, so all results and findings are only describing this topic from an athlete's perspective. Additionally, all the athletes included in this research were based in the UK and Ireland and their views and opinions may not be representative of the worldwide athlete community.

## Areas for future research

There is a need to explore the integration of implementation science into sports nutrition to better understand how to optimize intervention delivery and individual tailoring. Future research should seek to develop and explore dynamic tailoring interventions that aggregate data from multiple sources, including digital devices, that have the ability to deliver individualized, contextualized and timely support to athletes (Riley et al., 2011; Chevance et al., 2020). Additionally, research efforts focusing on ideating, developing and validating algorithms that can help automate certain sports nutrition tasks may be worth exploring in an attempt to help practitioners scale their service delivery in a time and cost-effective manner. During these processes it is recommended that that the acceptability of any novel applications, as well as athletes' engagement with these technologies, is explored (Perski et al., 2016; Perski and Short, 2021).

## Conclusion

These findings advance our understanding of the current issues surrounding communication strategies in applied sports nutrition, as well as identifying future opportunities for mobile apps to support practitioners' service provision. Specifically, this study identified that athletes experience a hybrid human-computer approach to nutrition support that they perceive lacks personalization from practitioners. In addition to increasing practitioner knowledge, time and availability to address this problem, additional training may be required to upskill in areas such as mobile technology as these digital tools now appear to afford sports nutritionists with new opportunities to develop scalable solutions and automated tools that may improve the individual tailoring an athlete receives in a time and cost-effective manner.

## Data availability statement

The original contributions presented in the study are included in the article/Supplementary material, further inquiries can be directed to the corresponding author.

## Ethics statement

The studies involving human participants were reviewed and approved by University Research Ethics Committee, Liverpool John Moores University. Written informed consent to participate in this study was provided by the participants' legal guardian/next of kin.

## Author contributions

All authors contributed to the design and implementation of the research, to the analysis of the results and to the writing of the manuscript.

## Conflict of interest

Authors DD, BC, SI, and CL-L are shareholders of Applied Behavior Systems Ltd. Authors JM and GC are shareholders of Pillar App Ltd. The remaining authors declare that the research was conducted in the absence of any commercial or financial relationships that could be construed as a potential conflict of interest.

## Publisher's note

All claims expressed in this article are solely those of the authors and do not necessarily represent those of their affiliated organizations, or those of the publisher, the editors and the reviewers. Any product that may be evaluated in this article, or claim that may be made by its manufacturer, is not guaranteed or endorsed by the publisher.

## References

[B1] Ahmed O. H. Weiler R. Schneiders A. G. McCrory P. Sullivan S. J. (2015). Top tips for social media use in sports and exercise medicine: doing the right thing in the digital age. Br. J. Sports Med. 49, 909–910. 10.1136/bjsports-2014-094395 25940634

[B2] Almirall D. Nahum-Shani I. Sherwood N. E. Murphy S. A. (2014). Introduction to SMART designs for the development of adaptive interventions: with application to weight loss research. Transl. Behav. Med. 4, 260–274. 10.1007/s13142-014-0265-0 25264466 PMC4167891

[B3] Bentley M. R. N. Mitchell N. Backhouse S. H. (2020). Sports nutrition interventions: a systematic review of behavioural strategies used to promote dietary behaviour change in athletes. Appetite. 150, 104645. 10.1016/j.appet.2020.104645 32112958

[B4] Bentley M. R. N. Patterson L. B. Mitchell N. Backhouse S. H. (2021). Athlete perspectives on the enablers and barriers to nutritional adherence in high-performance sport. Psychol. Sport Exerc. 52, 101831. 10.1016/j.psychsport.2020.101831 31124393

[B5] Braun V. Clarke V. (2006). Using thematic analysis in psychology. Qual. Res. Psychol. 3, 77–101. 10.1191/1478088706qp063oa

[B6] Braun V. Clarke V. (2013). Successful Qualitative Research: A Practical Guide For Beginners. London: Sage.

[B7] Braun V. Clarke V. (2019). Reflecting on reflexive thematic analysis. Qual. Res. Sport Exerc. Health. 11, 589–597. 10.1080/2159676X.2019.1628806

[B8] Browne J. D. Boland D. M. Baum J. T. Ikemiya K. Harris Q. Phillips M.. (2021). Lifestyle modification using a wearable biometric ring and guided feedback improve sleep and exercise behaviors: a 12-month randomized, placebo-controlled study. Front. Physiol. 12, 777874. 10.3389/fphys.2021.777874 34899398 PMC8656237

[B9] Burke L. M. Hawley J. A. (2018). Swifter, higher, stronger: what's on the menu? Science. 362, 781–787. 10.1126/science.aau2093 30442803

[B10] Chevance G. Perski O. Hekler E. B. (2020). Innovative methods for observing and changing complex health behaviors: four propositions. Transl. Behav. Med. 11, 676–685. 10.1093/tbm/ibaa026 32421196 PMC7963282

[B11] Close G. L. Hamilton D. L. Philp A. Burke L. M. Morton J. P. (2016). New strategies in sports nutrition to increase exercise performance. Free Radic. Biol. Med. 98:144–158. 10.1016/j.freeradbiomed.2016.01.016 26855422

[B12] Close G. L. Kasper A. M. Morton J. P. (2019). From paper to podium: quantifying the translational potential of performance nutrition research. Sports Med. 49, 25–37. 10.1007/s40279-018-1005-2 30671902 PMC6445818

[B13] Collins J. Maughan R. J. Gleeson M. Bilsborough J. Jeukendrup A. Morton J. P.. (2020). UEFA expert group statement on nutrition in elite football. Current evidence to inform practical recommendations and guide future research. Br. J. Sports Med. 55, 416–442. 10.1136/bjsports-2019-101961 33097528

[B14] Craig K. J. T. Morgan L. C. Chen C.-H. Michie S. Fusco N. Snowdon J. L.. (2020). Systematic review of context-aware digital behavior change interventions to improve health. Transl. Behav. Med. 11, 1037–1048. 10.1093/tbm/ibaa099 33085767 PMC8158169

[B15] Crawford M. J. (2002). Systematic review of involving patients in the planning and development of health care. BMJ. 325, 263–1263. 10.1136/bmj.325.7375.1263 12458240 PMC136920

[B16] Dearing J. W. Cox J. G. (2018). Diffusion of innovations theory, principles, and practice. Health Affairs. 37, 183–190. 10.1377/hlthaff.2017.1104 29401011

[B17] Deloitte (2020). Connected Health: How digital technology is transforming health and social care. Deloitte Centre for Health Solutions. Available online at: https://www2.deloitte.com/content/dam/Deloitte/uk/Documents/life-sciences-health-care/deloitte-uk-connected-health.pdf

[B18] Dunne D. M. Lefevre C. Cunniffe B. Tod D. Close G. L. Morton J. P.. (2019). Performance nutrition in the digital era–an exploratory study into the use of social media by sports nutritionists. J. Sports Sci. 37, 2467–2474. 10.1080/02640414.2019.1642052 31345110

[B19] Eccles M. P. Mittman B. S. (2006). Welcome to implementation science. Implement. Sci. 1, 1. 10.1186/1748-5908-1-1

[B20] Falter M. Budts W. Goetschalckx K. Cornelissen V. Buys R. (2019). Accuracy of apple watch measurements for heart rate and energy expenditure in patients with cardiovascular disease: cross-sectional study. JMIR mHealth and uHealth. 7, e11889. 10.2196/11889 30888332 PMC6444219

[B21] Foo W. L. Faghy M. A. Sparks A. Newbury J. W. Gough L. A. (2021). The effects of a nutrition education intervention on sports nutrition knowledge during a competitive season in highly trained adolescent swimmers. Nutrients. 13, 2713. 10.3390/nu13082713 34444873 PMC8400374

[B22] Gagnon K. Sabus C. (2014). Professionalism in a digital age: opportunities and considerations for using social media in health care. Phys. Ther. 95, 406–414. 10.2522/ptj.20130227 24903111

[B23] Gertler M. S. (2003). Tacit knowledge and the economic geography of context, or the undefinable tacitness of being (there). J. Econ. Geogr. 3, 75–99. 10.1093/jeg/3.1.75

[B24] Goetz L. H. Schork N. J. (2018). Personalized medicine: motivation, challenges, and progress. Fertil. Steril. 109, 952–963. 10.1016/j.fertnstert.2018.05.006 29935653 PMC6366451

[B25] Gratton C. Jones I. (2004). Research Methods for sport studies. London: Routledge.

[B26] Hardeman W. Houghton J. Lane K. Jones A. Naughton F. (2019). A systematic review of just-in-time adaptive interventions (JITAIs) to promote physical activity. Int. J. Behav. Nutr. Phys. Act. 16, 31. 10.1186/s12966-019-0792-7 30943983 PMC6448257

[B27] Heaney S. O'Connor H. Michael S. Gifford J. Naughton G. (2011). Nutrition knowledge in athletes: a systematic review. Int. J. Sport Nutr. Exerc. Metab. 21, 248–261. 10.1123/ijsnem.21.3.248 21719906

[B28] Huckvale K. Venkatesh S. Christensen H. (2019). Toward clinical digital phenotyping: a timely opportunity to consider purpose, quality, and safety. npj Digital Medicine. 2, 88. 10.1038/s41746-019-0166-1 31508498 PMC6731256

[B29] Impey S. G. Hearris M. A. Hammond K. M. Bartlett J. Louis J. Close G. L.. (2018). Fuel for the work required: a theoretical framework for carbohydrate periodization and the glycogen threshold hypothesis. Sports Med. 45, 1031–1048. 10.1007/s40279-018-0867-7 29453741 PMC5889771

[B30] Jeukendrup A. E. (2017). Periodized Nutrition for Athletes. Sports Med. 47, 51–63. 10.1007/s40279-017-0694-2 28332115 PMC5371625

[B31] Johnson K. B. Wei W. Weeraratne D. Frisse M. E. Misulis K. Rhee K.. (2020). Precision medicine, AI, and the future of personalized health care. Clin. Transl. Sci. 14, 86–93. 10.1111/cts.12884 32961010 PMC7877825

[B32] Jonvik K. L. King M. Rollo 1. Stellingwerff T. Pitsiladis Y. (2022). New opportunities to advance the field of sports nutrition. Front. Sports Act. Living. 4, 1–8. 10.3389/fspor.2022.852230 35252862 PMC8891369

[B33] Kitzinger J. (1995). Qualitative research: introducing focus groups. Br. Med. J. 311, 299–302. 10.1136/bmj.311.7000.299 7633241 PMC2550365

[B34] Kye B. Han N. Kim E. Park Y. Jo S. (2021). Educational applications of metaverse: possibilities and limitations. J. Educ. Eval. Health prof. 18, 32. 10.3352/jeehp.2021.18.32 34897242 PMC8737403

[B35] Masys D. R. (2002). Effects of current and future information technologies on the health care workforce. Health Aff. 21, 33–41. 10.1377/hlthaff.21.5.33 12224907

[B36] McGee J. B. Begg M. (2008). What medical educators need to know about “Web 2.0.” Med. Teach. 30, 164–169. 10.1080/01421590701881673 18464141

[B37] McKay A. K. A. Stellingwerff T. Smith E. S. Martin D. T. Mujika I. Goosey-Tolfrey V. L.. (2022). Defining training and performance caliber: a participant classification framework. Int. J. Sports Physiol. Perform. 29, 1–15. 10.1123/ijspp.2021-0451 34965513

[B38] Miller D. J. Roach G. D. Lastella M. Scanlan A. T. Bellenger C. R. Halson S. L.. (2021). A validation study of a commercial wearable device to automatically detect and estimate sleep. Biosensors. 11, 185. 10.3390/bios11060185 34201016 PMC8226553

[B39] Nahum-Shani I. Smith S. N. Spring B. J. Collins L. M. Witkiewitz K. Tewari A.. (2017). Just-in-time adaptive interventions (JITAIs) in mobile health: key components and design principles for ongoing health behavior support. Ann. Behav. Med. 52, 446–462. 10.1007/s12160-016-9830-8 27663578 PMC5364076

[B40] Perski O. Blandford A. West R. Michie S. (2016). Conceptualising engagement with digital behaviour change interventions: a systematic review using principles from critical interpretive synthesis. Transl. Behav. Med. 7, 254–267. 10.1007/s13142-016-0453-1 27966189 PMC5526809

[B41] Perski O. Short C. E. (2021). Acceptability of digital health interventions: embracing the complexity. Transl. Behav. Med. 11, ibab048. 10.1093/tbm/ibab048 33963864 PMC8320880

[B42] Plews D. J. Scott B. Altini M. Wood M. Kilding A. E. Laursen P. B. (2017). Comparison of heart-rate-variability recording with smartphone photoplethysmography, polar H7 Chest Strap, and Electrocardiography. Int. J. Sports Physiol. Perform. 12, 1324–1328. 10.1123/ijspp.2016-0668 28290720

[B43] Reeder B. David A. (2016). Health at hand: a systematic review of smart watch uses for health and wellness. J. Biomed. Inform. 63, 269–276. 10.1016/j.jbi.2016.09.001 27612974

[B44] Riley W. T. Rivera D. E. Atienza A. A. Nilsen W. Allison S. M. Mermelstein R. (2011). Health behavior models in the age of mobile interventions: are our theories up to the task? Transl. Behav. Med. 1, 53–71. 10.1007/s13142-011-0021-7 21796270 PMC3142960

[B45] Shaw A. (2019). Analysis: Premiership squads and homegrown percentages for 2019/20. www.rugbypass.com. Available online at: https://www.rugbypass.com/news/analysis-premiership-squads-and-homegrown-percentages-for-2019-20/

[B46] Sladdin I. Ball L. Bull C. Chaboyer W. (2017). Patient-centred care to improve dietetic practice: an integrative review. J. Hum. Nutr. Diet. 30, 453–470. 10.1111/jhn.12444 28124489

[B47] Smith B. McGannon K. R. (2017). Developing rigor in qualitative research: problems and opportunities within sport and exercise psychology. Int. Rev. Sport Exerc. Psychol. 11, 101–121. 10.1080/1750984X.2017.1317357

[B48] Sparkes A. C. Smith B. (2014). Qualitative Research Methods in Sport, Exercise and Health: From Process to Product. London: Routledge. Available online at: https://www.routledge.com/Qualitative-Research-Methods-in-Sport-Exercise-and-Health-From-Process/Sparkes-Smith/p/book/9780415578356

[B49] Steinhubl S. R. Muse E. D. Topol E. J. (2015). The emerging field of mobile health. Sci. Transl. Med. 7, 283rv3–283rv3. 10.1126/scitranslmed.aaa3487 25877894 PMC4748838

[B50] Stellingwerff T. Bovim I. M. Whitfield J. (2019a). Contemporary nutrition interventions to optimize performance in middle-distance runners. Int. J. Sport Nutr. Exerc. Metab. 29, 106–116. 10.1123/ijsnem.2018-0241 30299184

[B51] Stellingwerff T. Morton J. P. Burke L. M. (2019b). A framework for periodized nutrition for athletics. Int. J. Sport Nutr. Exerc. Metab. 29, 141–151. 10.1123/ijsnem.2018-0305 30632439

[B52] Tam R. Flood V. M. Beck K. L. O'Connor H. T. Gifford J. A. (2021). Measuring the sports nutrition knowledge of elite Australian athletes using the Platform to Evaluate Athlete Knowledge of Sports Nutrition Questionnaire. Nutr. Diet. 78, 535–543. 10.1111/1747-0080.12687 34155780

[B53] Tausch A. P. Menold N. (2016). Methodological aspects of focus groups in health research. Global Qual. Nurs. Res. 3, 1–12. 10.1177/2333393616630466 28462326 PMC5342644

[B54] Thomas D. T. Erdman K. A. Burke L. (2016). American college of sports medicine joint position statement. Nutrition and athletic performance. Med. Sci. Sports Exerc. 48, 543–568. 10.1249/MSS.0000000000000852 26891166

[B55] Tong A. Sainsbury P. Craig J. (2007). Consolidated criteria for reporting qualitative research (COREQ): a 32-item checklist for interviews and focus groups. Int. J. Qual. Health Care. 19, 349–357. 10.1093/intqhc/mzm042 17872937

[B56] Turner S. J. (2010). Qualitative interview design: a practical guide for novice investigators. Qual. Rep. 15, 754–760. 10.46743/2160-3715/2010.1178

[B57] Vandelanotte C. Müller A. M. Short C. E. Hingle M. Nathan N. Williams S. L.. (2016). Past, present, and future of eHealth and mhealth research to improve physical activity and dietary behaviors. J. Nutr. Educ. Behav. 48, 219–228.e1. 10.1016/j.jneb.2015.12.006 26965100

[B58] Walsh N. P. (2019). Nutrition and athlete immune health: new perspectives on an old paradigm. Sports Med. 49, 153–168. 10.1007/s40279-019-01160-3 31691927 PMC6901425

[B59] Wang L. Miller L. C. (2019). Just-in-the-moment adaptive interventions (JITAI): a meta-analytical review. Health Commun. 35, 1531–1544. 10.1080/10410236.2019.1652388 31488002

[B60] Wong L. P. (2008). Focus group discussion: a tool for health and medical research. Singapore Med. J. 49, 256–260. 18363011

