# Supplementary material for: Athlete experiences of communication strategies in applied sports nutrition and future considerations for mobile app supportive solutions
Source: Front Sports Act Living. 2022 Sep 12;4:911412. doi: 10.3389/fspor.2022.911412 (PMC9512279; doi:10.3389/fspor.2022.911412)
Supplement: Supplementary file 1 [file Data_Sheet_1.docx]

**Appendix 1**

*Focus Group Interview Guide*

| Objectives | Key Questions |
| --- | --- |
| 1. To gather athletes’ perceptions and thoughts of current communication strategies and performance nutrition support in elite sport. | - Can you describe the current performance nutrition support you receive, if any, in terms of how it is delivered (for example format, frequency, contact time)? Tell me more. What are your thoughts on this? - What do you think of the current performance nutrition support provided to you in terms of how it is delivered? Can you describe your level of satisfaction? Why? - What do you think of the current support provided to you in terms of information that is delivered? Can you describe your level of satisfaction? From your perspective how might the information be improved? - What do you think of the current support provided to you in terms of resources and how they are delivered? Can you describe your level of satisfaction? From your perspective how might the information be improved? - How would you describe the level of mental challenge you experience with respect to the resources and information that is delivered to you? - How would you describe the level of physical challenge, such as cooking and shopping, you experience with respect to the resources and information that is delivered to you? |
| 2. To attain preliminary opinions and views on the use of mobile apps in practice. | - Have you received any support via a mobile phone? If so, can you describe that? If not, is this something that you feel may be beneficial? - Are you aware of any personalized mobile apps that deliver performance nutrition support? Can you describe your experience of interacting with these? - What is your opinion of personalized mobile apps to help deliver performance nutrition support? How could this help your nutrition behaviours? Why or why not? - Do you think having this option could benefit you? Why or why not? In what situations specifically? - What are your thoughts on remote one-to-one’s? Would you take part? Why or why not? |
| 3. To identify athletes preferred app features and types of content. | - What would drive you to open a nutrition app? For example, emotions such as boredom. - How frequently do you experience this? - What would the most important features of this app be to you? For example, stories, viewing peers’ profiles, saving content, ability to gain followers, recipes, and calorie counters. - What features would you have to have? Why? - What types of content would you prefer, for example, video, picture, text? - Should content be tailored to your level of knowledge? For example, individuals with low levels of knowledge would receive more simple and basic content than someone has a high level of knowledge. How would this look? |
