# Supplementary material for: Athlete experiences of communication strategies in applied sports nutrition and future considerations for mobile app supportive solutions
Source: Front Sports Act Living. 2022 Sep 12;4:911412. doi: 10.3389/fspor.2022.911412 (PMC9512279; doi:10.3389/fspor.2022.911412)
Supplement: Supplementary file 2 [file Data_Sheet_2.docx]

**Appendix 2** *Communication strategies & information delivery*

| **Raw Data** | **Sub Theme** | **Higher Order Theme** |
| --- | --- | --- |
| “Last year when I needed a bit more help, I was on a nutrition app, so I’d send the nutritionist every meal and even just pictures. This was easy for me.” *(Athlete 1, Focus Group 1)* |  | Communication strategies & information delivery *(n= 33)* |
| “It was good to have that conversation with the nutritionist (as a result of submitting food pictures to them via a mobile nutrition app) and for them to be like ‘oh why don’t you try swapping this for this’ or like suggestions in terms of that. So, it was useful” *(Athlete 1, Focus Group 1)* |  |  |
| “We used an app (Meal Logger) which I took pictures of my food and we’d send it to the nutritionist every day. The nutritionist was just seeing how I was eating, what I was eating, when I was eating and then I’d use another app (myfitnesspal) which scanned bar codes of whatever you were eating and whatever you were making and you’d put it in and it’d count your calories” *(Athlete 1, Focus Group 1)* |  |  |
| “the nutritionist is always at the end of a phone or a WhatsApp, which is really handy” *(Athlete 4, Focus Group 1)* |  |  |
| “I spoke to the nutritionist on Instagram and got a few things which I felt like I was lacking” *(Athlete 1, Focus Group 2)* |  |  |
| “on the Instagram page, like there are enough avenues for us to get it (nutrition education) … I think there will be more chance of them picking it up than an e-mail” *(Athlete 2, Focus Group 2)* |  |  |
| “WhatsApping, there’s loads and loads of nutrition support” *(Athlete 2, Focus Group 2)* |  |  |
| “I know I can message the nutritionist (over WhatsApp) anyway if I was short of a recipe” *(Athlete 3, Focus Group 2)* |  |  |
| “I think by having a nutritionist here all the time is easier because then you can just grab them in passing and be like ‘this is what I’ve eaten. Quite often when I weigh in, in the morning we’ll chat about what I’ve eaten over the last day and why I’m heavy or why I’ve lost weight and where I can look at targeting to help put that on. I think that’s been really good” *(Athlete 3, Focus Group 2)* |  |  |
| “The nutritionist is here most of the time, but I probably only really speak to them seriously about nutrition maybe once every 3 weeks. That would be about right. I think it’s got better this year. I think last year you could not have really had a proper conversation for a couple of months at a time” *(Athlete 1, Focus Group 2)* |  |  |
| “the nutritionist is available to talk to but obviously it’s limited contact time. They give us talks when we’re in (international) camp... they’re not always at every camp. And obviously if they are there and everyone’s trying to get some input from them, you can’t sit down for an hour and discuss things" *(Athlete 2, Focus Group 1)* |  |  |
| “I think the nutritionist is not in all the time but when they are in, it's like once a week...I say, we have a meeting once a month” *(Athlete 1, Focus Group 3)* |  |  |
| “We’ve had stuff sent on WhatsApp which helps…PDF documents and nutrition plans” *(Athlete 2, Focus Group 3)* |  |  |
| “it’s a bit brief, the nutritionist gives us a powerpoint on the basics we need to know, like carbs, the intakes and stuff” *(Athlete 4, Focus Group 3)* |  |  |
| “It’s (the nutrition presentation) just very basic, you can’t individualise for all of us” *(Athlete 1, Focus Group 4)* |  |  |
| “The S&C coach does a presentation, but I don’t think they've even done it this year.” *(Athlete 1, Focus Group 4)* |  |  |
| “I think that online support would be a game changer, especially for women in football because we’re so far behind. I feel we’re far behind. That’s the thing, players probably do want to ask questions and if you do ask a sports scientist sometimes, they don’t actually have the nutritionist answer” *(Athlete 3, Focus Group 4)* |  |  |
| “they always WhatsApp, ‘if you ever need anything just give us a shout’. So, it’s very much in our hands. There’s only so much they can tell us really and it’s about us relaying it to them” *(Athlete 1, Focus Group 5)* |  |  |
| “I think using apps are ideal really because everyone’s on their phone aren’t they” *(Athlete 1, Focus Group 5)* |  |  |
| “We had some nutrition at the start of the year on training camp, we did some tests and went over a few things there in a bit of a presentation and a similar thing in February, and it’s just mainly going through, what you’d do on different days in terms of how much training you’re doing. So, in terms of amounts and stuff, mainly, I think what I took from it was like your protein fat level stays the same pretty much every day but its mainly, carb intake was like the important thing.” *(Athlete 1, Focus Group 5)* |  |  |
| “If we’ve got a question the nutritionist will reply (via message) within an hour or something” *(Athlete 2, Focus Group 5)* |  |  |
| “what you need to eat on certain days, what you need to eat around match days, after matches and the nutritionist sends out e-mails with examples of finger food.” *(Athlete 1, Focus Group 7)* |  |  |
| “if you ask to see the nutritionist, it’s whenever you’re injured. They’ll say, "we’ll have a little meeting" but then they'll give you stuff and I think everyone had a meeting in pre-season to go over everything” *(Athlete 2, Focus Group 7)* |  |  |
| “There's a WhatsApp group for nutrition...the nutritionist will put loads of stuff in, like some days they’ll say there’s going to be an update on what’s going to be in the canteen that day, on what type of thing you can eat and what type of day you’ve had and if there’s a game they’ll say what you should be eating” *(Athlete 2, Focus Group 7)* |  |  |
| “The nutritionist sends e-mails and stuff like that” *(Athlete 4, Focus Group 7)* |  |  |
| “Yeah they were thorough so that’s why the contact time wasn’t regular because you did take away quite a lot of information from one but it was sometimes a bit overwhelming, I would say, and I was like ‘crap I don’t do any of this” *(Athlete 1, Focus Group 8)* |  |  |
| “I’ve spoken to the nutritionist a few times and we’ve had WhatsApp conversations when I’ve been in America, just to check, on a few of the things that we’ve agreed to do…having WhatsApp conversations are handy because you can literally send a picture or have a chat about a recipe or something quite quickly” *(Athlete 1, Focus Group 8)* |  |  |
| “I’d definitely probably go and see the nutritionist every couple of weeks, I think. I didn’t think they were any good so I used to see someone else. In 2016, I saw someone else for a while, and that was probably a couple of times a year but like was in contact via e-mail and WhatsApp and things.” *(Athlete 2, Focus Group 8)* |  |  |
| “it (the nutrition support) was basically working so that I could get in touch with the nutritionist over WhatsApp if I had any concerns, mainly to do with anti-doping and like supplementations and stuff like that” *(Athlete 2, Focus Group 8)* |  |  |
| “I probably saw the nutritionist, spoke to them properly over Skype in that time and I had a really big chat with them but then I didn’t speak to them again in-between those two chats” *(Athlete 3, Focus Group 8)* |  |  |
| “it just feels like the same, everything, the powerpoints, the whole thing, it’s the same. We’ve seen it for five years” *(Athlete 2, Focus Group 9)* |  |  |
| “the same Powerpoints that we’ve been seeing for quite a few years” *(Athlete 3, Focus Group 9)* |  |  |
| “We need a bit more individualisation. The Powerpoints are for the average, everyone’s different so it might help one person but not help the rest of the group so I think if we just follow that it might not be benefiting us” *(Athlete 7, Focus Group 9)* |  |  |
|  |  |  |

**Appendix 3** *Acceptance and adoption of the online practitioner*

| **Raw Data** | **Sub Theme** | **Higher Order Theme** |
| --- | --- | --- |
| “The nutritionist is always on the end of a phone or a WhatsApp, which is really handy.” *(Athlete 4, Focus Group 1)* |  | Acceptance and adoption of the online practitioner *(n=26)* |
| “I just want to be able to message, the nutritionist ‘what are your thoughts on this?” *(Athlete 3, Focus Group 2)* |  |  |
| “I think that online support would be a game changer, especially for women in football because we’re so far behind. I feel we’re far behind. That’s the thing, players probably do want to ask questions and if you do ask a sports scientist sometimes, they don’t actually have the nutritionist answer.” *(Athlete 3, Focus Group 4)* |  |  |
| “they're quite frequent, to be fair, and they always WhatsApp, ‘if you ever need anything just give us a shout’ which is good. So, it’s very much in our hands. There’s only so much they can tell us really and it’s about us relaying it to them.” *(Athlete 1, Focus Group 5)* |  |  |
| “If we’ve got a question the nutritionist will reply (via message) within an hour or so which helps” *(Athlete 3, Focus Group 5)* |  |  |
| “I believe every day’s a school day. You can never know enough... so if it’s handy for me to pick up the phone and ask someone who is far more advanced than what I am, ask them a question and get their opinion then obviously that would benefit every athlete I think.” *(Athlete 3, Focus Group 6)* |  |  |
| “having WhatsApp conversations are handy because you can literally send a picture or have a chat about a recipe or something quite quickly” *(Athlete 1, Focus Group 8)* |  |  |
| “Last year when I needed a bit more help, I was on a nutrition app, so I’d send the nutritionist every meal and even just pictures. That was easy for me.” *(Athlete 1, Focus Group 1)* |  |  |
| “it was good to have that conversation with the nutritionist (as a result of submitting food pictures to them via a mobile nutrition app) and for them to be like ‘oh why don’t you try swapping this for this’ or like suggestions in terms of that. So, it was useful.” *(Athlete 1, Focus Group 1)* |  |  |
| “I’ve spoken to the nutritionist a few times and we’ve had WhatsApp conversations when I’ve been in America, just to check, on a few of the things that we’ve agreed to do” *(Athlete 1, Focus Group 8)* |  |  |
| “WhatsApping, there’s loads and loads of nutrition support” *(Athlete 2, Focus Group 2)* |  |  |
| “on the Instagram (club nutrition) page, like there are enough avenues for us to get it (nutrition education)” *(Athlete 2, Focus Group 2)* |  |  |
| “I know I can message the nutritionist (over WhatsApp) anyway if I was short of a recipe which is really helpful'” *(Athlete 3, Focus Group 2)* |  |  |
| “We’ve had stuff sent on WhatsApp which helps…PDF documents and nutrition plans” *(Athlete 2, Focus Group 3)* |  |  |
| “There's a WhatsApp group for nutrition...the nutritionist will put loads of stuff in, like some days they’ll say there’s going to be an update on what’s going to be in the canteen that day, on what type of thing you can eat and what type of day you’ve had and if there’s a game they’ll say what you should be eating” *(Athlete 2, Focus Group 7)* |  |  |
| “I spoke to the nutritionist on Instagram and got a few things which I felt like I was lacking” *(Athlete 1, Focus Group 2)* |  |  |
| “it (the nutrition support) was basically working so that I could get in touch with the nutritionist over WhatsApp if I had any concerns” *(Athlete 2, Focus Group 8)* |  |  |
| “It would work better if there was a coach on the other side (of an app)” *(Athlete 3, Focus Group 5)* |  |  |
| “I think it (remote one-to-one's) would be good, like, a weekly thing, to have a chat with the nutritionist and just make sure that you’re staying on top of it.” *(Athlete 4, Focus Group 5)* |  |  |
| “I’d be asking you questions all the time ‘what about this, would that benefit me?’ *(Athlete 5, Focus Group 6)* |  |  |
| “if there was someone there, working with me every day and at the end of the day you will ask them won’t you*.” (Athlete 5, Focus Group 6)* |  |  |
| “I’d find that (remote one-to-one's) useful.” *(Athlete 1, Focus Group 8)* |  |  |
| “In case of a certain situation, we should be able to contact the nutritionist any time” *(Athlete 2, Focus Group 9)* |  |  |
| “I’d prefer remote coaching compared to on-site support” *(Athlete 3, Focus Group 9)* |  |  |
| “I think, in passing it’s easy at the Club but having an app is much easier. You can just be like, ‘boom’ rather than be like ‘come and I’ll see you at this time’ and you’re ‘well actually I can’t see you at that time’ or you’ve got to change everything around it. It’s hard enough anyway when you’re trying to book in to see a coach or something”  *(Athlete 1, Focus Group 2)* |  |  |
| “I think using apps are ideal really because everyone’s on their phone aren’t they*” (Athlete 1, Focus Group 5)* |  |  |
|  |  |  |

**Appendix 4** *A personalization problem*; *Limited contact time*

| **Raw Data** | **Sub Theme** | **Higher Order Theme** |
| --- | --- | --- |
| “the nutritionist is available to talk to but obviously it’s limited contact time. They give us talks when we’re in (international) camp... they’re not always at every camp. And obviously if they are there and everyone’s trying to get some input from them, you can’t sit down for an hour and discuss things” *(Athlete 2, Focus Group 1)* | Limited contact time  *(n= 17)* | A personalization problem *(n= 47)* |
| “I think just because of the limited time the nutrition support is quite generic... it is better to have a bit more input on an individual basis” *(Athlete 2, Focus Group 1)* |  |  |
| “we know that there’s somebody there who can help us with nutrition but not everybody knows that” (*Athlete 5, Focus Group 1)* |  |  |
| “I don’t know how much they get paid, whether they’re full time or what but they don’t put in the effort to go in to that much detail. Whether that’s because they don’t have the time or because they don’t get paid enough. I’m not saying they don’t have the knowledge but if you’re going to go through 45 players and say ‘right, your macros are this’ or ‘your target is this and this is why …’, no-one’s going to do to that from my perspective.” *(Athlete 5, Focus Group 1)* |  |  |
| “the one thing I would say is, because I’ve been injured quite a lot, when it is the day where the match day/fit boys aren’t in, it (the nutrition support) kind of goes down a bit” *(Athlete 2, Focus Group 2)* |  |  |
| “I think the nutritionist is not in all the time but when they are in, it's like once a week...I say, we have a meeting once a month” *(Athlete 1, Focus Group 3)* |  |  |
| “it’s a bit brief, the nutritionist gives us a powerpoint on the basics we need to know, like carbs, the intakes and stuff” *(Athlete 4, Focus Group 3)* |  |  |
| “I think it would have been good sometimes if the nutritionist came on a race with us and in stage racing be, like, ‘maybe stay away from this food because today was a flatter day’ or maybe try and get a bit more specific each day. I think there could have been more of that, to sort of tell us more specific stuff when we’re racing. Obviously, that’s when we need it more” *(Athlete 3, Focus Group 5)* |  |  |
| “it’s different with First Team and the U23s. The First Team gets loads of nutrition support, literally everything, and the 23’s, we just get, we get some supplements.” (*Athlete 1, Focus Group 7)* |  |  |
| “if you ask to see the nutritionist, it’s whenever you’re injured. They’ll say, "we’ll have a little meeting" but then they'll give you stuff and I think everyone had a meeting in pre-season to go over everything.” (*Athlete 2, Focus Group 7)* |  |  |
| “If I had to know something, sometimes I just Google it and get the answer quite easily.” (*Athlete 2, Focus Group 7)* |  |  |
| “I think we get told a lot (individually), a couple of times throughout the season.” (*Athlete 4, Focus Group 7)* |  |  |
| “Yeah the nutritionist was thorough so that’s why the contact time wasn’t regular because you did take away quite a lot of information from one meeting but it was sometimes a bit overwhelming, I would say, and I was like ‘crap I don’t do any of this’” (*Athlete 1, Focus Group 8)* |  |  |
| “I get probably low contact time with the nutritionists” (*Athlete 1, Focus Group 8)* |  |  |
| “I was only really given access to the nutritionist when I was injured, so there was a problem and then I got sent to the nutritionist which wasn’t very productive” (*Athlete 3, Focus Group 8)* |  |  |
| “You shouldn’t have to pester the person to help you” (*Athlete 3, Focus Group 9)* |  |  |
| “Any sort of communication’s better than what it is at the moment.” (*Athlete 7, Focus Group 9)* |  |  |

**Appendix 5** *A personalization problem*; *Lack of personalization*

| **Raw Data** | **Sub Theme** | **Higher Order Theme** |
| --- | --- | --- |
| “it (the nutrition support) was only in terms of ideas really but it’s not really player specific stuff.” (*Athlete 1, Focus Group 1)* | Lack of personalization  *(n= 32)* | A personalization problem |
| “people don’t individually know what their goal should be. Say if you’re in fat club, people don’t know what weight they need to get to?!” *(Athlete 1, Focus Group 1)* |  |  |
| “It’s (the nutrition support) not really player specific stuff” *(Athlete 2, Focus Group 1)* |  |  |
| “I think just because of the limited time the nutrition support is quite generic... it is better to have a bit more input on an individual basis” *(Athlete 2, Focus Group 1)* |  |  |
| “It’s the same in the national team, our S&C, they think it’s like ‘oh you need to be more than 85 kilos’ and I was like ‘do you know I’m 70 at the moment?’ but that’s unrealistic.” *(Athlete 2, Focus Group 1)* |  |  |
| “It’s (the nutrition support) just a bit too generic” *(Athlete 3, Focus Group 1)* |  |  |
| “I follow like a macro specific diet and I found that really worked but we don’t get offered anything in that much detail.” *(Athlete 5, Focus Group 1)* |  |  |
| “The only thing I’d probably say, which I’ve found across nutritionists, not just our current one, it’s all the same thing in terms of the recipe side of it. It’s all well and good having some extra oats or whatever before bed but it’s all just the same things that we’re eating. How can we make it a bit more inventive to actually enjoy our food a little bit more?” *(Athlete 5, Focus Group 1)* |  |  |
| “I don’t know how much they get paid, whether they’re full time or what but they don’t put in the effort to go in to that much detail (individual macro and calorie targets). Whether that’s because they don’t have the time or because they don’t get paid enough. I’m not saying they don’t have the knowledge but if you’re going to go through 45 players and say ‘right, your macro is this’ or ‘your macro is this and this is why …’, no-one’s going to do to that from my perspective” *(Athlete 5, Focus Group 1)* |  |  |
| “If you had specific goals you were focusing on and you had a plan set in place...That you could literally follow day in, day out, everyone would follow it.” (*Athlete 1, Focus Group 9)* |  |  |
| “If you could narrow it (a nutrition plan) down to your personal needs then it would be beneficial.” *(Athlete 6, Focus Group 9)* |  |  |
| “I think for me because I asked for some help on certain stuff, it was like there was information there but to me it was like, I’ve got the information but I’m not really going to follow it because there’s not enough information, if you get what I mean?” *(Athlete 1, Focus Group 3)* |  |  |
| “I think it’s hard because the nutritionist has got to do the whole squad so they can’t just individualise it for everyone” *(Athlete 3, Focus Group 3)* |  |  |
| “it’s a bit brief, the nutritionist gives us a powerpoint on the basics we need to know, like carbs, the intakes and stuff” *(Athlete 4, Focus Group 3)* |  |  |
| “There’s a basic structure there but there’s nothing, I wouldn’t say, in-depth or anything” *(Athlete 4, Focus Group 4)*  “It’s (the nutrition support) just very basic, you can’t individualise, for all of us” *(Athlete 1, Focus Group 4)* |  |  |
| “the nutrition support is and there is too much for you to personally go away and try and work it out.” *(Athlete 1, Focus Group 4)* |  |  |
| “The S&C coach does a presentation, but I don’t think they've even done it this year.” *(Athlete 1, Focus Group 4)* |  |  |
| “You could go to the S&C coach and they could try and help you but they're just trying to generalise everything rather than the detail that could help” *(Athlete 2, Focus Group 4)* |  |  |
| “Actually, knowing when and what you should take or how much on what days, is poor, as in you don’t really know” *(Athlete 2, Focus Group 4)* |  |  |
| “What I’m saying is we’re all told to eat the same sort of thing and I think that’s quite hard.” *(Athlete 3, Focus Group 4)* |  |  |
| “we’ve done testing before, last season we did fat testing and stuff and then there was a fat burning exercise regime implemented which was like walking on treadmills for 45 minutes before training to burn X amount of calories to put you in a deficit but it was never about the nutrition side” *(Athlete 3, Focus Group 4)* |  |  |
| “the S&C coach follows his own stuff (nutrition advice) which might not work for somebody else” *(Athlete 5, Focus Group 4)* |  |  |
| “the nutrition support is just a general assumption as to what your training was, like medium, heavy day, etc. It’s just a general assumption, it wasn’t more specific to say what you needed” *(Athlete 1, Focus Group 5)* |  |  |
| “I think what the nutritionist does is pretty much pointless I’d say. It should be related to exactly what your training is, and it should be completely personal. Unless it’s every day with your training and then related to that it’s pointless *(Athlete 2, Focus Group 5)* |  |  |
| “you hear so many different things and you try different things. You don’t know what, and it seems to be changing all the time. Like some people say ‘strip fat, don’t eat any fat’, that type of thing and others are like ‘smash it and reduce carbs’ and that type of thing. So you never know if what you’re doing is best for you. Because I imagine for some people it should be different for individuals, but it’s just knowing, finding a way to find what really works” *(Athlete 3, Focus Group 5)* |  |  |
| “Quite repetitive…very very not satisfied (with the nutrition support)” *(Athlete 1, Focus Group 9)* |  |  |
| “it just feels like the same, everything, the powerpoints, the whole thing, it’s the same. We’ve seen it for five years” *(Athlete 2, Focus Group 9)* |  |  |
| “the same Powerpoints that we’ve been seeing for quite a few years” *(Athlete 3, Focus Group 9)* |  |  |
| “I’ve been given, one list (if nutrition advice), I haven’t really got it individualised, it’s more a list that everyone gets” *(Athlete 4, Focus Group 9)* |  |  |
| “We need a bit more individualisation. The Powerpoints are for the average, everyone’s different so it might help one person but not help the rest of the group so I think if we just follow that it might not be benefiting us” *(Athlete 7, Focus Group 9)* |  |  |
| “We just get the general guidelines and we don’t get told certain sections that we need to start focusing on” *(Athlete 8, Focus Group 9)* |  |  |

**Appendix 6** *Tailoring Technology; Periodized and personalized nutrition plans*

| **Raw Data** | **Sub Theme** | **Higher Order Theme** |
| --- | --- | --- |
| “I think, being really general, professional sports men and women like structure and a plan, in general, you always get your odd ones that don’t... if you give us a structure that we can follow, a personal plan, then I think you probably get better results over a period of time.” *(Athlete 2, Focus Group 2)* | *Periodized and personalized nutrition plans (n=36)* | Tailoring Technology *(n=54)* |
| “you know when we have the schedule out in pre-season and the nutritionist was like ‘have a high carb meal or a low carb meal during the day’, I found that very helpful.” *(Athlete 3, Focus Group 2)* |  |  |
| “I just think knowing what you’re eating, like when I say ‘oh, I’m not eating too much’, just knowing that what you’re actually eating is going to benefit you because it is ultimately for your performance and you as an individual.” *(Athlete 1, Focus Group 4)* |  |  |
| “I think it will benefit us having a food plan...we had to plan yesterday what we’re eating today so knowing what I should pack with me just covering my two days.” *(Athlete 2, Focus Group 4)* |  |  |
| “The thing I find quite important, and I think Training Peaks do that quite well, when you’ve got your week up and then you can have a look at a weekly snapshot and stuff. So yeah, I suppose the layout is quite important (for nutrition plans)” *(Athlete 2, Focus Group 8)* |  |  |
| “if I’ve got an off day then I’ve got another day that I’ve got my four or five sessions, what is the difference? What do I need to be doing?” *(Athlete 1, Focus Group 1)* |  |  |
| “I think just tracking and making sure I’m consistently getting the right amount of food at the right time and it fitting in with training. So, if I’ve got a light week because I’m rehabbing then I’m having the right food, if trainings heavier then I’m having the right food but mainly just making sure I am eating enough every day” *(Athlete 2, Focus Group 1)* |  |  |
| “It would be great if you had an app where you could write ‘right, this is what I’m doing this week, we are on our training programs’ and then if they said ‘right, this is how many macros you need’ or whatever, for that workout for that day and week and if you’re not doing that much, ‘this is how much, how many calories you need and have it all been broken down’. So, flipping it on its head with inputting training and then knowing what to eat” *(Athlete 5, Focus Group 1)* |  |  |
| “I don’t know if this is incorporated but if you had an app where you have your periodization and you click on meals and say you had just a general one and you thought ‘right I need a red breakfast, amber, etc’ and you click on that and get some recipes and then almost put the recipe into a basket but instead of the recipe being in there, the ingredients and then you can literally just go to that at the end of your week and once you’ve done your week and be ‘right, that’s everything I need to buy and have to go and get it’, that would be awesome.” *(Athlete 1, Focus Group 2)* |  |  |
| “where he was saying about the decision making, it takes it out of it because you’re like ‘oh, it’s Tuesday afternoon, I know I’ve picked that recipe’. That’s what me and my partner do. We go through a Meal in 15 cookbook with that carb periodization plan and we are just choosing it and then just doing one shop and then we know what I need to eat” *(Athlete 3, Focus Group 2)* |  |  |
| “Those carb periodization frameworks would be useful and I think with recipes that go with it. So, if you are saying something like a low carb or something like that, just be like ‘this is a great option, this is easy to do, boom, there’s the recipe” *(Athlete 3, Focus Group 2)* |  |  |
| “the nutritionist gave me the nutrition plan, it was all colour and it was literally breakfast, snack, lunch, snack, tea, snack before bed. I was building but I look at that and I think ‘right, that’s just a load of words on the page’ so, what we’ve agreed to do is have green for when the meals are high carb and then, red when it’s high protein...so when I look at it I know ‘right that’s green, that’s gonna be a high carb meal’ so then I can pick out in the week when it’s gonna be and especially, the high carbs the day before games” *(Athlete 1, Focus Group 3)* |  |  |
| “you can link a colour coded plan to, here’s a high carb meal, a video of a high carb meal that you could have match day -1 or something so if you linked that” *(Athlete 2, Focus Group 3)* |  |  |
| “Being given some of the scales, you know, the ones on the wall at the moment, saying if it’s a light session have so much carbohydrates, so much protein” *(Athlete 4, Focus Group 3)* |  |  |
| “we literally just want basic, like, what can we eat, how much of it should we be having and, on a training day, on a non-training day. It depends on how active we are.” *(Athlete 1, Focus Group 4)* |  |  |
| “I think it (a periodized nutrition plan) is better…it seems easier” *(Athlete 2, Focus Group 4)* |  |  |
| “if you go in in the morning and you’ve got a hard day, most people would still just have a couple of eggs and then think that they can go and do, you know, an intense session. So the fact that now they’re (the national team) saying ‘actually you need this amount of carbs, you need this amount of whatever’, I think, for me personally, it helps because I would just go and have the same lunch and dinner” *(Athlete 3, Focus Group 4)* |  |  |
| “nutrition is easier when you’re in camp than it is when you’re here at the club although the club should be because we’re together more but they have, so through the week they’ll have our training schedule...if it’s a hard day they’ll have, now next to the food they’ll have, green, amber, red and,, they’ll have a plate so when you go in they’ll have a plate and how much more, so if it’s two red and one green that’s what they advise you to have” *(Athlete 3, Focus Group 4)* |  |  |
| “It seems easier, if you know what colour food you are” *(Athlete 4, Focus Group 4)* |  |  |
| “Today was savage and then, you know, a few days ago wasn’t as hard so it would be interesting to see if that would differ, change what we eat” *(Athlete 1, Focus Group 5)* |  |  |
| “What about a match up thing, say you put in what type of day you’re going to have and then it comes up with pictures of different scenarios of what you could eat at certain times and then you took a picture and try and match it up to what it should be” *(Athlete 1, Focus Group 5)* |  |  |
| “if you can see the type of thing, rather than, taking the time to put every little ingredient in you’ve got, you can see what it needs to roughly look like in size and stuff and you try and like make it” *(Athlete 2, Focus Group 5)* |  |  |
| “I think nutrition should be planned, you know at the start of the season, you plan your races and that, it should be planned out, in week blocks, almost like your training is, like you train hard for this long and then you taper and then, like, with your weight and stuff like that. So, like a long-term general plan and then, like, every week your training gets a daily specific diet” *(Athlete 3, Focus Group 5)* |  |  |
| “The most important feature would be a nutrition weekly calendar” *(Athlete 3, Focus Group 4)* |  |  |
| “What if there was like a training program, for the amount of meals that a person had” *(Athlete 1, Focus Group 6)* |  |  |
| “Maybe someday I’ll probably be using an app just to, you know, because you can see, if you’ve had a hard day, what you could see what sort of things you should eat. You just give it the information and it makes a decision for you” *(Athlete 4, Focus Group 7)* |  |  |
| “Or it promotes a recipe like ‘this is an example of a high protein one that you could use’. Do you know this menu? No. Okay and have a list of things, like one side carb, one side protein. My day’s high or low and to kind of know when to slot it in” *(Athlete 2, Focus Group 8)* |  |  |
| “What would be cool is if you could do something based on what training you put in and what you should be eating” *(Athlete 3, Focus Group 8)* |  |  |
| “saying what’s your intensity of the day? Just for Women, like what your training’s been like in that day so you can find out and match it with the diet or nutrition” *(Athlete 3, Focus Group 8)* |  |  |
| “I’m thinking about everyone’s day’s different so constructing the whole day as a quite flexible app, because some days you double run and some days you do gym sessions and running and it’s, the sports are different as well.” *(Athlete 3, Focus Group 8)* |  |  |
| “Like if it’s on a red day (high intensity) you should be allowed to have more of certain things, do you know what I mean?” *(Athlete 4, Focus Group 9)* |  |  |
| “It’d be nice also to have recipes that are grouped so if I’m searching, I want a recipe that has these macros rather than searching through all the recipes” (*Athlete 1, Focus Group 1)* |  |  |
| “I’d like to see more ideas about calorie targets, but also give me some nice meals that I can make” *(Athlete 2, Focus Group 1)* |  |  |
| “You could colour co-ordinate your recipes so it could say, hard or easy or whatever. So, if I’m scrolling through and it looks really nice and it says ‘easy’ I’ll think ‘oh I’m gonna click on that then” *(Athlete 3, Focus Group 3)* |  |  |
| “I would say that it would be nice to suggest recipes, because I know there are certain things you can have that are anti-inflammatory and if it’s tailored for athletes that would be really useful” *(Athlete 1, Focus Group 8)* |  |  |
| “That would be the main thing I’d go for, the ideas, recipe ideas, what meals to cook” *(Athlete 2, Focus Group 3)* |  |  |
| “I wanna be able to cook and learn how to know what to cook” *(Athlete 4, Focus Group 9)* |  |  |

**Appendix 7** *Tailoring Technology; Goal setting, monitoring and feedback*

| **Raw Data** | **Sub Theme** | **Higher Order Theme** |
| --- | --- | --- |
| “if you knew what those (nutrition) goals were and if you had a benchmark it would be better” *(Athlete 5, Focus Group 1)* | *Goal setting, monitoring and feedback*  *(n=14)* | Tailoring Technology *(n=54)* |
| “If you had specific goals you were focusing on and you had a plan set in place...That you could literally follow day in, day out, everyone would follow it.” *(Athlete 1, Focus Group 9)* |  |  |
| “If you could narrow it (nutrition goals) down to your personal needs then it would be beneficial.” *(Athlete 6, Focus Group 9)* |  |  |
| “Yeah MyFitnessPal was (useful) especially when I was doing hard days, I realised I wasn’t eating anywhere near enough” *(Athlete 1, Focus Group 5)* |  |  |
| “it’s (MyFitnessPal) making you aware of what you’re eating but you’re not getting anything out of it other than the information. It’s not telling you ‘this will help you more, this will help you more” *(Athlete 3, Focus Group 3)* |  |  |
| “I’ve tried MyFitnessPal but I didn’t use calories, we changed the customise idea to carb intake, so I changed it for my training and stuff. I did it for a bit and it seemed to work, I just got bored with it eventually putting everything in every day” *(Athlete 1, Focus Group 5)* |  |  |
| “the reason I bought LifeSum is that I’m more inclined to use it as I find it much easier to input your own recipes so I can actually cook and make what I want and actually have all the macros and everything” *(Athlete 1, Focus Group 3)* |  |  |
| “setting goals and seeing how far off you are and when you’re hitting those, so if I’ve got a goal for the end of September, how far off am I? With your FitBit and it’s like ‘tick, you’ve eaten all your calories” *(Athlete 1, Focus Group 1)* |  |  |
| “if you could like take pictures, like progress pictures that would be useful.” *(Athlete 2, Focus Group 1)* |  |  |
| “Even in an app, inputting a bit of personal information would be useful, so you can actually track your weight and record things so you can see if it is actually making a difference...So say if you are 70kg on this date and you use this and you can actually see a difference, ‘oh I’m actually 68.4 now’ and you can see that” *(Athlete 2, Focus Group 6)* |  |  |
| “It’s like, with training peaks that we use for training, you go on that after you’ve done your training to see how it went, that type of thing, if it’s going good or shit or, looking for PBs, that type of thing I want, to see how it went*.” (Athlete 1, Focus Group 5)* |  |  |
| “Inputting data, like on Training Peaks, like every few days we do a weigh in and stuff like that, it should have something like that where you do your weight and what else can you do… and then you can have graphs and see how you’re doing, sort of thing” *(Athlete 2, Focus Group 5)* |  |  |
| “an app would need to kind of give you challenges and stuff and something to keep you wanting to go back to it” *(Athlete 2, Focus Group 6)* |  |  |
| “For me, it’d be quite nice just to see a little green tick, yeah, a little way of knowing you’ve hit your critical target...knowing you’ve done all you can in that day.” *(Athlete 2, Focus Group 8)* |  |  |

**Appendix 8** *Tailoring Technology; Notifications and reminders*

| **Raw Data** | **Sub Theme** | **Higher Order Theme** |
| --- | --- | --- |
| “if you’ve got a notification, like so and so has smashed the day, see what they’ve eaten” *(Athlete 1, Focus Group 1)* | *Notifications and reminders (n=7)* | Tailoring Technology *(n=54)* |
| “I think, to be honest, maybe, for me, a reminder, you know it could notify you because, like on days off especially, I can go through and I can be hungry but not cook because I can’t be bothered to get out of bed and that’s genuine*.” (Athlete 1, Focus Group 2)* |  |  |
| “if, say, it pings up a reminder and it’s like boom ‘have this meal’ and then I’m like, okay fine, ten minutes cooking and then it’s done” *(Athlete 1, Focus Group 2)* |  |  |
| “I found notifications very good, just a reminder ‘did you have 5 portions of fruit and veg today?’ and you’re like ‘shit I haven’t.” *(Athlete 4, Focus Group 1)* |  |  |
| “It would be good to have notifications...like, when to eat” *(Athlete 1, Focus Group 5)* |  |  |
| “it feels like a notification that came up with daily facts, that popped up on my screen and was like ‘did you know pineapple apparently decreases inflammation" you know what I mean, little facts, you would open it up, you would read it” *(Athlete 1, Focus Group 6)* |  |  |
| “alerts and stuff like that would be helpful, different things to keep you engaged with the app. Following a path, you know, would be good.” *(Athletes 4, Focus Group 6)* |  |  |
|  |  |  |

**Appendix 9** *Tailoring Technology; Performance focused content*

| **Raw Data** | **Sub Theme** | **Higher Order Theme** |
| --- | --- | --- |
| “I think (in app information needs) a performance focus” *(Athlete 1, Focus Group 1)* | *Performance focused content (n=8)* | Tailoring Technology *(n=54)* |
| “I would like to have a bit of a reason why you’re doing the nutrition plan” *(Athlete 4, Focus Group 1)* |  |  |
| “I think information is the most important thing… If there were no videos, I wouldn’t really bother to be honest” *(Athlete 5, Focus Group 4)* |  |  |
| “the knowledge about food, I think that would be really important, if I had, say if I had an app there and you click on it and it will tell you ‘well beetroot will open up your arteries and help blood flow’ and things like that, then I’ll add a bit of beetroot into my food” *(Athlete 3, Focus Group 6)* |  |  |
| “I bet there’s loads of knowledge out there that certain foods help you in different situations, if you’re sore this food will help me for this. ‘I’ve got a really hard training session coming up, I need to be lighter’, ‘this would be the correct food to have’, do you know what I mean? If an app had that sort of knowledge I would use it every single day, yeah, every day” *(Athlete 3, Focus Group 6)* |  |  |
| “I think that an app should be in detail and might have at the start be quite simple so that everyone understands and then maybe underneath you might have the more complicated details of it because if you really know what you want to do or what you’re eating things for then that would be the reason why.” *(Athlete 2, Focus Group 7)* |  |  |
| “And there should be somewhere recipes link to know more about the little bit of why” *(Athlete 3, Focus Group 8)* |  |  |
| “I would say that it would be nice to suggest recipes, because I know there are certain things you can have that are anti-inflammatory and if it’s tailored for athletes that would be really useful” *(Athlete 1, Focus Group 8)* |  |  |
|  |  |  |
