# Supplementary material for: Athlete experiences of communication strategies in applied sports nutrition and future considerations for mobile app supportive solutions
Source: Front Sports Act Living. 2022 Sep 12;4:911412. doi: 10.3389/fspor.2022.911412 (PMC9512279; doi:10.3389/fspor.2022.911412)
Supplement: Supplementary file 3 [file Data_Sheet_3.docx]

**Biographical note**

David M. Dunne is a PhD researcher within the Research Institute of Sport and Exercise Sciences at Liverpool John Moores University and performance nutritionist with applied experience working with Harlequins Rugby, British Canoe, European Tour and Queens Park Rangers Football Club. His research focuses on developing optimal digital practices for sports nutrition practitioners.

Carmen E. Lefevre is a behavioral scientist and honorary senior research associate at UCL's Centre for Behavior Change. Her work spans theoretical and applied work into health and wellbeing, from understanding the effects of Instagram on Orthorexia to studying the appearance benefits of fruit and vegetable consumption.

Brian Cunniffe is a head of performance support at the English Institute of Sport, where he leads science and medical provision to a number of Olympic and Paralympic athletes. He has an honorary research position with University College London and is an active researcher in applied sports sciences and innovation spanning a number of domains.

Samuel G. Impey is a sports scientist whose academic research has focused on carbohydrate metabolism and the interaction of training and nutrition, Sam currently holds an adjunct lecture position at Edith Cowen University in Perth. In addition to his academic work Sam has supported professional athletes over the last 10 years with a focus on endurance sports.

David Tod is a senior lecturer in sport psychology and has over 100 scientific publications.  His research focuses on expertise development in psychologists and effectiveness in applied sport psychology.  He has and continues to consult in professional sport and the performing arts.

James P. Morton is a professor of exercise physiology and has in excess of 100 publications and book chapters. His research interest focuses on carbohydrate and energy metabolism, with his research spanning across several sports including boxing and horse racing. He co-leads the MSc Sport and Exercise Nutrition program at Liverpool John Moores University and is currently the head of performance solutions at Science in Sport.

Graeme Close co-leads the MSc Sport and Exercise Nutrition program at Liverpool John Moores University and has authored over 100 papers and book chapters with multiple focusing on professional jockeys and weight making. He is a nutrition advisor to England Rugby.

Rebecca C. Murphy is Subject Head in the School of Sport and Exercise Sciences at Liverpool John Moores University. Her research background focuses on developing education and behavior change interventions.
